# Enhancement of hypoxia-activated prodrug TH-302 anti-tumor activity by Chk1 inhibition

**DOI:** 10.1186/s12885-015-1387-6

**Published:** 2015-05-21

**Authors:** Fanying Meng, Deepthi Bhupathi, Jessica D Sun, Qian Liu, Dharmendra Ahluwalia, Yan Wang, Mark D Matteucci, Charles P Hart

**Affiliations:** Threshold Pharmaceuticals, 170 Harbor Way, Suite 300, South San Francisco, CA 94080-6108 USA

**Keywords:** Cancer, Hypoxia, Chk1 inhibitor, Hypoxia-activated prodrug, DNA damage, DNA repair, *in vitro* cytotoxicity, *in vivo* anti-tumor activity, Xenograft models

## Abstract

**Background:**

The hypoxia-activated prodrug TH-302 is reduced at its nitroimidazole group and selectively under hypoxic conditions releases the DNA cross-linker bromo-isophosphoramide mustard (Br-IPM). Here, we have explored the effect of Chk1 inhibition on TH-302-mediated pharmacological activities.

**Methods:**

We employed *in vitro* cell viability, DNA damage, cellular signaling assays and the *in vivo* HT29 human tumor xenograft model to study the effect of Chk1inhibition on TH-302 antitumor activities.

**Results:**

TH-302 cytotoxicity is greatly enhanced by Chk1 inhibition in p53-deficient but not in p53-proficient human cancer cell lines. Chk1 inhibitors reduced TH-302-induced cell cycle arrest via blocking TH-302-induced decrease of phosphorylation of histone H3 and increasing Cdc2-Y15 phosphorylation. Employing the single-cell gel electrophoresis (comet) assay, we observed a potentiation of the TH-302 dependent tail moment. TH-302 induced γH2AX and apoptosis were also increased upon the addition of Chk1 inhibitor. Potentiation of TH-302 cytotoxicity by Chk1 inhibitor was only observed in cell lines proficient in, but not deficient in homology-directed DNA repair. We also show that combination treatment led to lowering of Rad51 expression levels as compared to either agent alone. *In vivo* data demonstrate that Chk1 inhibitor enhances TH-302 anti-tumor activity in p53 mutant HT-29 human tumor xenografts, supporting the hypothesis that these *in vitro* results can translate to enhanced *in vivo* efficacy of the combination.

**Conclusions:**

TH-302-mediated *in vitro* and *in vivo* anti-tumor activities were greatly enhanced by the addition of Chk1 inhibitors. The preclinical data presented in this study support a new approach for the treatment of p53-deficient hypoxic cancers by combining Chk1 inhibitors with the hypoxia-activated prodrug TH-302.

## Background

Hypoxia in solid tumors and the affected bone marrow of hematologic malignancies is a prevalent feature of cancer. Cells in the hypoxic tumor microenvironment are more resistant to radiotherapy and to most antiproliferative cancer drugs, and also acquire a more malignant and metastatic phenotype [1]. One therapeutic approach being developed for the treatment of cancer is hypoxia-activated cytostatic or cytotoxic prodrugs [2].

TH-302 is a hypoxia-activated prodrug of bromo-isophosphoramide (Br-IPM) that is reduced at its 2-nitroimidazole group and selectively activated under the severe hypoxic conditions commonly found in tumors, but not typically observed in normal tissues [3]. Br-IPM is a potent DNA alkylating agent, and kills tumor cells by creating DNA crosslinks [4]. Preclinical data demonstrate that TH-302 exhibits anti-tumor activity both as a monotherapy as well as in combination with other cancer therapies [5-7]. Clinically, TH-302 has been investigated in several early stage trials [8-11] and is currently being evaluated in Phase III trials in soft-tissue sarcoma in combination with doxorubicin and pancreatic cancer in combination with gemcitabine (NCT01440088 and NCT01746979, respectively).

There are two major cell-cycle checkpoint systems for detecting and responding to DNA damage: the G_1_/S and intra-S checkpoints system to prevent the replication of damaged DNA, and the G_2_/M checkpoint to prevent segregation of damaged chromosomes. The majority of tumors are deficient in the G_1_/S DNA damage checkpoint due to tumor suppressor p53 mutations. Pharmacological inhibition of the remaining intact G_2_/M checkpoint, e.g. through Chk1 inhibition, should lead to enhanced tumor cell death, as compared with p53 proficient normal tissue [12]. It has been shown that inhibition of Chk1 signaling using small molecule inhibitors, dominant negative constructs, interference RNA (RNAi), or ribozymes leads to abrogation the G_2_/M checkpoint, impaired DNA repair, sensitization of p53-deficient cells to apoptosis, and an increase in tumor cell death [13-15]. Of particular note, Chk1 inhibitors have also been designed as prodrugs for selective activation in the hypoxic regions of tumors [15,16].

Chk1 also regulates homology-directed repair (HDR), as DNA damage-induced HDR is dependent on Chk1-mediated Rad51 phosphorylation. Chk1 inhibition leads to impaired Rad51 foci formation, a key step in HDR [17,18]. Abrogation of Chk1 function leads to persistent unrepaired DNA double-strand breaks (DSBs). Chk1 inhibition results in premature mitotic entry in response to DNA damaging agents thus resulting in increased phosphorylated histone H3, a marker of mitosis [19]. In addition, Chk1 pathway plays an important role in protecting cells from caspase-3-mediated apoptosis [20,21]. Reports have shown that cells with reduced levels of Chk1 were found to be more prone to apoptosis [14,21,22]. More recently, it has been reported that Chk1 may have prognostic and predictive significance in breast cancer [23].

Chk1 inhibition can potentiate the cytotoxicity of radiation and genotoxic therapies [24-29]. Chk1 inhibitors have been widely studied and a select number of compounds have reached early clinical trials. Notable among these are the ATP-competitive inhibitors LY2603618, PF477736, AZD7762, SCH90077617, and LY260636818 [5], the latter three of which have progressed to Phase II clinical trials. Here we describe the combination therapeutic efficacy profile of Chk1 inhibitors with the hypoxia-activated prodrug TH-302 in *in vitro* and *in vivo* preclinical models.

## Methods

### Reagents and cell lines

TH-302 was from Syngene, AZD7762 and LY2603618 were from Selleck Chemicals, and PF477736 was from Tocris Bioscience. RIPA was from Sigma. Protease inhibitor cocktails were from Thermo Scientific. ChemiGlow substrate was from Proteinsimple. ECL reagent, Rad51 mAb, actin mAb, goat anti-rabbit HRP, goat anti-mouse HRP, and cell cycle reagents were from EMD Millipore. γH2AX mAb was from Epitomics. Antibodies against phospho-Histone H3, phospho-Cdc2 Y15 antibody, total Chk1, phospho-Chk1 (S296) were from Cell Signaling. Total Cdc2 p34 antibody was from Santa Cruz Biotechnology. FITC-conjugated goat anti-mouse secondary antibody and AlamarBlue cell viability reagent were from Life Technologies. Comet assay kit was from Trevigen. Caspase Glo 3/7 assay system was from Promega. Isogenic p53 proficient and deficient cell line pairs were from Horizon Discovery. All other cell lines were from ATCC.

### *In vitro* proliferation

Exponentially growing cells were seeded 24 h prior to addition of test compounds. After compound addition, the plates were incubated for 2 h under either normoxia (21% O_2_) or hypoxia (N_2_) supplied with 5% CO_2_ at 37°C. After wash, cells were cultured for an additional 70 h in fresh medium containing Chk1 inhibitors under normoxia (21% O_2_), and the viable cells were quantified using either AlamarBlue or ATP assay and normalized using either vehicle for single treatment or Chk1 inhibitor alone for combination treatment. IC_50_ was calculated using Prism software. The level of synergism by the Chou-Talalay method was expressed as the Combination Index (CI) calculated using CalcuSyn software [30].

### Cell cycle analysis

Cells were treated with 0.1 μM of either PF477736 or AZD7762 and TH-302 for 2 h under either normoxia (21% O_2_) or hypoxia (N_2_). Following wash, cells were cultured for additional 22 h in the presence of Chk1 inhibitor under normoxia. Cells were fixed in 75% ethanol and cell cycle distribution was determined using Cell Cycle reagent and Guava flow cytometry (EMD Millipore).

### Single cell gel electrophoresis comet assay

After seeding cells for 24 h, TH-302 and 0.1 μM of AZD7762 were added and incubated for 24 h under either normoxia (21% O_2_) or hypoxia (0.1% O_2_). For cross-linking assessment experiments, cells were treated with 20 μM of bleomycin for 1 h starting at the end of the TH-302 treatment period. Comet assay was performed with Trevigen’s single-cell electrophoresis system. The data was analyzed using Comet Assay IV software from Perceptive Instruments.

### Detection of γH2AX

HT29 colon cancer cells were treated with vehicle or TH-302 for 2 h under either normoxia (21% O_2_) or hypoxia (N_2_) conditions with or without 0.1 μM of AZD7762, and then continuously incubated for additional 4 h in the presence of AZD7762 for the combination group and AZD7762 monotherapy group. Cells were permeabilized with 1% Triton X-100 and incubated with γH2AX monoclonal antibody for 2 h and goat anti-mouse-FITC for 1 h. Cells were imaged using a Nikon TS-100 fluorescent microscope.

### Caspase activity

HT-29 cells were exposed to TH-302 and 0.1 μM of AZD7762 for 2 h under either normoxia (21% O_2_) or hypoxia (N_2_). After wash, cells were continuously cultured for additional 46 h in the presence of 0.1 μM of AZD7762. Luminescence-based caspase activity assay was performed based on the manufacturer’s (Promega) instructions.

### Western blot

HT29 cells were exposed to TH-302, AZD7762, or combined TH-302 and AZD7762 for 2 h under either normoxia (21% O_2_) or hypoxia (N_2_). After removal of TH-302, cells were continuously incubated with AZD7762 for additional 46 h. Cell extracts were prepared and protein concentrations were determined. Proteins were detected after SDS-PAGE and Western blotting with ChemGlow detection system (ProteinSimple) using antibodies recognizing autophospho-Chk1 (S296), total Chk1, phospho-Histone H3, phosphorylated Cdc2 Y15, total Cdc2, Rad51, and actin.

### *In vivo* antitumor activity

Female nude mice (4-6 weeks; Nu-Foxn 1^nu^ NU/NU, Charles River Laboratories) were tagged with microchips (Locus Technology) for identification. All animal studies were approved by the Institutional Animal Care and Use Committee at Threshold Pharmaceuticals.

HT29 cells were mixed with 50% Matrigel and 0.2 ml/mouse were subcutaneously implanted to the flank area of the animals. When the tumor size reached 150 mm^3^, mice were randomized into experimental groups.

TH-302 was dissolved in saline (0.9% NaCl) at 5 mg/ml and filtered prior to animal dosing. AZD7762 was formulated in 1% DMSO, 11.3% cyclodextrin in water for injection. Maximum tolerated dose (MTD) for TH-302 in combination with AZD7762 was determined in a small number of non-tumor bearing nu/nu mice. The MTD was defined as the highest possible dose resulting in no animal deaths, less than 20% weight loss for any one animal in an experimental group, no significant changes in general clinical signs, and no abnormal gross anatomical findings after necropsy. The doses of compounds used in all studies were no higher than MTD.

Two dosing regimens of TH-302 were employed in the study. With the TH-302 intermittent dosing regimen, TH-302 was dosed intraperitoneally (ip) at 100 mg/kg, twice/wk x 2 wks. AZD7762 was dosed i.p. at either 20 or 12.5 mg/kg, four times/wk x 2 wks. For the combination therapy study, two different dosing sequences were investigated: (1) TH-302 was given first, and 4 h and 24 h later AZD7762 was administered (the ‘TAA’ sequence); and (2) AZD7762 was given first, and 4 h and 24 h later followed by TH-302 and AZD7762, respectively (the ‘ATA’ sequence). With the TH-302 daily dosing regimen, TH-302 was dosed ip at 50 mg/kg, with a regimen of QDx5/wk x 2wks. AZD7762 12.5 mg/kg was given under same regimen as TH-302. When the combination was scheduled, TH-302 was administered 4 h prior to AZD7762 (the ‘TA’ sequence).

Tumor growth and body weight were measured twice a week. Tumor volume was calculated as (length x width^2^)/2. Drug efficacy was assessed as Tumor Growth Inhibition (TGI) and Tumor Growth Delay (TGD). TGI was defined as (1-ΔT/ΔC) x 100, where ΔT/ΔC presented the ratio of the change in mean tumor volume of the treated group and of the control group. TGD was calculated as the extra days for the treated tumor to reach 1000 mm^3^ as compared to control group (TGD_1000_). Animals were culled when individual tumor size was over 2000 mm^3^ or individual tumor size was over 1000 mm^3^ if mean tumor volume exceeded 1000 mm^3^ in the group. Conditional survival was defined as the time that an animal reached the endpoint of a tumor size of 1000 mm^3^. Kaplan-Meier plots were constructed based on the percentage animals surviving in each group as a function of time. Median time (MT) is the time at which half the animals in the group had a tumor size less than 1000 mm^3^. The antitumor activity was evaluated as follows: T/C % = MT of treated group/MT of control group × 100. Results were also expressed as the percentage of increased life span (ILS, T/C of treated group–100). Statistical significance between the groups was evaluated by the log-rank test.

Data are expressed as the mean ± SEM. One-way analysis of variance with Dunnett post-comparison test (GraphPad PRISM 4) or two-tail student’s *t* test were used for analysis. A *P* level < 0.05 was considered statistically significant.

### Histology and immunohistochemistry

300-600 mm^3^ HT29 xenograft tumors were used in the pharmacodynamics studies. Six animals per group were treated with vehicle, AZD7762 25 mg/kg, ip, on Day 1 and Day 2, TH-302 150 mg/kg, ip on Day 1 or the combination of AZD7762 and TH-302. In the combination treatment, two dosing sequences were used: TAA and ATA (as above). Tumors were harvested on Day 3 (which was 24 h after the second AZD7762 treatment), and fixed in 10% neutral buffered formalin and embedded in paraffin. 5 μm thick paraffin sections were cut and adhered to poly-_L_-lysine-coated glass slides.

After deparaffinization and rehydration of the slides, antigen was retrieved. Endogenous peroxidase was quenched by Peroxidaze 1 and non-specific binding was blocked by Background Sniper (both Biocare Medical). Slides were incubated with rabbit monoclonal anti γH2AX (Epitomics, 1:3000), or rabbit polyclonal anti-phospho Chk1 (phospho S345, Chk1-S345, Abcam, 1:25) or Caspase 3 (Cell Signaling Technology, 1:300) for 1 h at RT followed by secondary HRP-conjugated anti rabbit IgG (Epitomics).

### Image analysis

γH2AX, phospho-Chk1-S345 or Caspase 3 positive cells were counted at 400x magnification. Ten fields per section were used. The percentage of positive cells was calculated as number of γH2AX or Chk1-S345 positive cells/number of total cells in the field x 100%. *P* value <0.05 was considered significant. One-way analysis of variance with Dunnett’s test (GraphPad PRISM 4) was used to compare the significance of the multiple groups. A student’s *t-test* was used to find the significance between two groups.

## Results

### Chk1 inhibitors sensitize human colon cancer HT29 cells to TH-302

**Figure 1:**
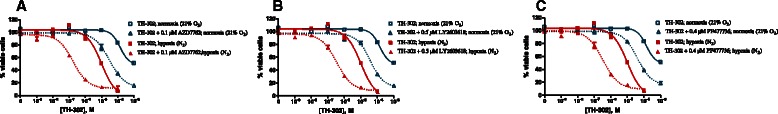
Enhanced TH-302 cytotoxicity by Chk1 inhibitors was observed in p53-mutant HT29 cells. HT29 cells were seeded for 24 h, and then exposed to TH-302 alone, or combined with 0.1 μM of AZD7762 **(A)**, 0.5 μM LY2603618 **(B)**, or 0.4 μM PF477736 **(C)** for 2 h under either air (21% O_2_) or hypoxia (N_2_). After wash, cells were treated with the Chk1 inhibitors for an additional 70 h under air (21% O_2_). The viable cells were detected using AlamarBlue and quantified with spectrophotometer. IC_50_ values are presented in Table 1. Combination indexes from Chou-Talalay (CalcuSyn) analyses are presented in Table 2 (air group and N_2_ group). The data are the representative of four independent experiments.

As shown in Figure 1A-C and Table 1, p53-deficient HT29 cells treated with TH-302 alone exhibited hypoxia-selective and concentration-dependent cytotoxicity (Table 1). The concentration of each Chk1 inhibitor selected for combination studies caused no significant effect on cell viability compared with vehicle-treated control cells. Combination of TH-302 and Chk1 inhibitors induced significant potentiation of the cytotoxic activity of TH-302 in HT29 cells. The combination index (CI) determined by the Chou-Talalay method was in a range of 0.9 to 0.001 (Table 2), thus ranging from demonstration of near additivity to very strong synergism.

**Table 1:** **Enhanced IC**_**50s**_ **were observed in combination groups**

	Normoxia (21% O_2_)	Hypoxia (N_2_)	HCR
	IC_50_(μM)	IC_50_(μM)	Normoxia/Hypoxia
TH-302	1000	10	100
AZD 7762	0.4	N/A	N/A
LY 2603618	1.7	N/A	N/A
PF 477736	0.8	N/A	N/A
TH-302 and 0.1 μM AZD	39	0.2	195
TH-302 and 0.5 μM LY	46	0.4	115
TH-302 and 0.4 μM PF	57	0.4	140

**Table 2:** **Near additivity or strong synergism was observed in combination groups**

Oxygen	TH-302 (μM)	Combination index (CI)		
		TH-302 + 0.1 μM AZD	TH-302 + 0.5 μM LY	TH-302 + 0.4 μM PF
Normoxia (21% O_2_)	0.001	0.001	0.001	0.001
	0.01	0.001	0.001	0.001
	0.1	0.02	0.04	0.01
	1	0.02	0.12	0.05
	10	0.24	0.18	0.17
	100	0.65	0.66	0.61
	1000	0.84	0.84	0.81
Hypoxia (N_2_)	0.0001	0.001	0.001	0.001
	0.001	0.001	0.001	0.001
	0.01	0.05	0.05	0.02
	0.1	0.36	0.25	0.23
	1	0.74	0.69	0.67
	10	0.86	0.86	0.84
	100	0.9	0.93	0.93

### Potentiation of TH-302 activity by Chk1 inhibitors is dependent on p53-deficiency

To explore the breadth of enhanced potency of TH-302 by the addition of Chk1 inhibitors, other human cell lines were also investigated. As shown in Table 3, the synergistic effects of TH-302 combined with Chk1 inhibitors were observed in the p53-deficient HeLa cell line but not in p53-proficient H460 or p53 heterozygous DU145 human tumor cell lines. To confirm the importance of p53 status in Chk1 inhibitor-mediated synergistic effect observed with TH-302, we employed the isogenic p53-proficient and -deficient cell line pairs, MCF10A p53^+/+^ and MCF10A p53^-/-^ and SW48 p53^+/+^ and SW48 p53^-/-^. Parental and p53-null MCF10A and SW48 cell lines exhibited a similar sensitivity to single agent AZD7762, LY2603618 and PF477736, regardless of the p53 status. TH-302 displayed hypoxia-selective and concentration-dependent cytotoxic activity that was comparable in both p53-proficient and -deficient cells. As expected, the combination treatment of TH-302 with the three Chk1 inhibitors yielded a trend of enhanced cytotoxicity profile in the cells that lack p53 compared with p53-proficient parental cells.

**Table 3:** **Enhanced TH-302 cytotoxicity by Chk1 inhibitors is only observed in p53-deficient but not p53-proficient cells**

Cell line	Cell type	p53 status	AZD 7762 (μM)	LY 2603618 (μM)	PF 477736 (μM)	TH-302 (Normoxia; 21% O_2_)				TH-302 (Hypoxia; N_2_)			
						-	+AZD 7762 (μM)	+LY 2603618 (μM)	+PF 477736 (μM)	-	+AZD 7762 (μM)	+LY 2603618 (μM)	+PF 477736 (μM)
HeLa	Cervical	p53 ^-/-^	0.7	3.2	0.3	>1000	95	90	200	100	0.5	0.4	2.1
H460	NSCLC	p53 ^+/+^	0.2	1.2	4.3	25	25	22	19	0.4	0.2	0.2	0.2
DU145	Prostate	p53 ^+/-^	0.4	2.3	0.7	210	80	110	340	2.7	1.1	0.9	1.9
MCF10A p53^+/+^	Breast	p53 ^+/+^	0.9	5.7	1	110	44	61	75	0.6	0.4	0.5	0.6
MCF10A p53^-/-^	Breast	p53 ^-/-^	0.5	4.1	0.7	88	24	22	34	0.7	0.1	0.2	0.2
SW48 p53^+/+^	Colon	p53 ^+/+^	0.1	4.1	0.4	39	16	35	39	0.09	0.04	0.07	0.2
SW48 p53^-/-^	Colon	p53 ^-/-^	0.1	0.6	0.1	110	19	22	27	1.5	0.2	0.2	0.2

IC_50_ values in μM. Inhibitor concentrations used for combination studies (all in μM): AZD7762: HeLa (0.2); H460 (0.05); DU145 (0.1); MCF10A p53^+/+^ and MCF10Ap53^-/-^(0.1); SW48 p53^+/+^ and SW48 p53^-/-^(0.03). LY2603618: HeLa (0.7); H460 (0.2); DU145 (0.4); MCF10A p53^+/+^ and MCF10Ap53^-/-^(1); SW48 p53^+/+^(0.5); SW48 p53^-/-^ (0.2). PF47736: HeLa (0.1); H460 (2); DU145 (0.1); 0.1 MCF10A p53^+/+^ and MCF10Ap53^-/-^(0.1); SW48 p53^+/+^ (0.2); SW48 p53^-/-^(0.05).

### Synergistic effect of AZD7762 and TH-302 is not dependent on the schedule of drug addition in HT29 cells

**Figure 2:**
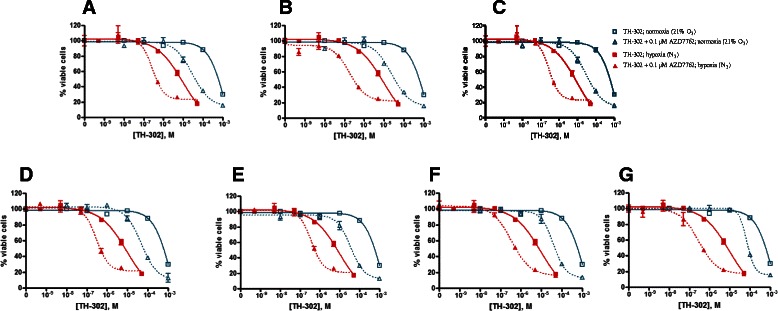
Enhanced TH-302 cytotoxicity by AZD7762 is not dependent on treatment schedule. HT29 cells were treated with TH-302 alone or combined with 0.1 μM of AZD7762, which was added 2 h before **(A)**, simultaneously **(B)**, or 0 h **(C)**, 2 h **(D)**, 4 h **(E)**, 6 h **(F)** or 24 h **(G)** after TH-302. TH-302 treatment time is 2 h at the indicated concentrations under either air (21% O_2_) or hypoxia (N_2_). Viable cells were quantified using the AlamarBlue assay. IC_50_ values are presented in Table 4. The data are the representative of two independent experiments.

To investigate whether the synergistic activity of the combination of AZD7762 and TH-302 is related to the sequence of drug addition, we conducted combination studies with various treatment schedules. As shown in Figure 2 and Table 4, addition of AZD7762 enhanced TH-302 activity regardless of dosing sequence or scheduling, which was evident with similar IC_50_ values with the different treatment schedules.

**Table 4:** **Similar IC**_**50s**_ **were observed in all combination groups regardless of treatment schedule**

	Normoxia (21% O_2_)	Potentiation	Hypoxia (N_2_)	Potentiation
	IC_50_(μM)	(fold)	IC_50_(μM)	(fold)
TH-302 only	580		7.4	
AZD7762 2 h before TH-302	39	15	0.4	19
AZD7762 with TH-302	34	17	0.3	25
AZD7762 0 h after TH-302	42	14	0.5	14
AZD7762 2 h post TH-302	61	10	0.4	19
AZD7762 4 h post TH-302	42	14	0.6	12
AZD7762 6 h post TH-302	46	13	0.6	12
AZD7762 24 h post TH-302	84	7	0.4	19

### AZD7762 reduces TH-302-induced cell cycle arrest

We then investigated the effect of Chk1 inhibitors on cell cycle progression in TH-302-treated cells. As shown in Table 5, HeLa cells treated with vehicle or PF477736 alone had no appreciable changes in cell cycle distribution as determined by DNA content analysis (flow cytometry). Treatment with TH-302 alone caused an accumulation of G_2_/M cells. Inhibition of Chk1 by PF47736 in cells treated with TH-302 reduced TH-302-mediated G_2_/M arrest under both normoxia and hypoxia.

**Table 5:** **Chk1 inhibitors reduced TH-302-mediated HeLa cell-cycle arrest**

	TH-302 μM	TH-302 alone			TH-302+0.1 μM PF		
		%G_0_/G_1_	%S	%G_2_/M	%G_0_/G_1_	%S	%G_2_/M
Normoxia (21% O_2_)	0	64	18	19	72	13	17
	12.5	65	11	24	65	13	23
	25	61	12	27	71	10	19
	50	31	17	52	57	14	28
Hypoxia (N_2_)	0.0625	75	8	17	71	12	18
	0.125	68	8	24	76	8	16
	0.25	54	10	36	60	11	29

We also investigated the effect of AZD7762 in HT29 cells under hypoxic (N_2_) conditions. As shown in Table 6, AZD7762 alone exhibited a minimal effect on cell cycle distribution, TH-302 alone induced concentration-dependent cell cycle arrest at G_2_/M, and at higher concentrations, TH-302 also induced a profound S phase arrest. The combination of TH-302 with 0.1 μM of AZD7762 caused a marked abrogation of TH-302-induced S-phase block. In the presence of AZD7762, increasing concentration of TH-302 up to 2 μM caused no changes in the S-phase population compared with the concentration-dependent increase of S-phase population observed in cells treated with TH-302 alone. Under normoxic (21% O_2_) conditions there were no changes of cell cycle distribution in HT29 cells treated with TH-302 at tested concentrations (data not shown).

**Table 6:** **Chk1 inhibitors reduced TH-302-mediated HT-29 cell-cycle arrest**

TH-302 μM	Hypoxia (N_2_)					
	TH-302			TH-302 + 0.1 μM AZD		
	%G_0_/G_1_	%S	%G_2_/M	%G_0_/G_1_	%S	%G_2_/M
0	53	21	25	62	19	19
0.125	36	17	47	45	17	37
0.25	25	15	59	40	15	42
0.5	17	20	62	28	19	49
1	15	30	54	22	19	55
2	14	51	33	24	18	53

### TH-302 and AZD7762 co-treatment induced DNA double-strand breaks, but not enhanced DNA cross-linking

**Figure 3:**
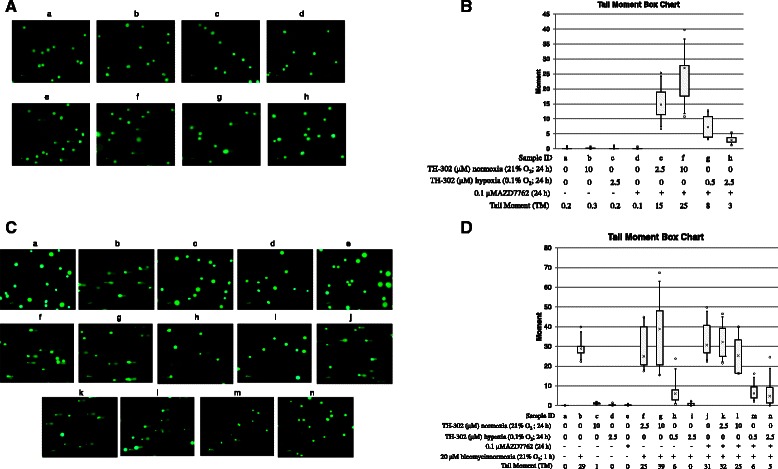
Co-treatment of TH-302 and AZD7762 increased tail moment but not cross-linking by Comet assay. After seeding HT-29 cells for 24 h, cells were treated at the indicated drugs and oxygen concentrations for 24 h. To assess DNA breaks, single cell gel electrophoresis comet assay was performed **(A)**. The length of tail moment (TM) was calculated using Comet Assay IV software **(B)**. For cross-linking assessment, cells were treated with 20 μM of bleomycin for 1 h at the end of the drug treatment, and then Comet assay was performed **(C)** and TM was calculated **(D)**. The data are the representative of three independent experiments.

To assess the impact of the Chk1 inhibitor AZD7762 on DNA double-strand breaks in TH-302 treated cells, we used the single-cell gel electrophoresis-based ‘comet’ assay. As shown in Figure 3A and B, cells treated with (a) vehicle, (b) TH-302 under normoxia (21% O_2_), (c) TH-302 under hypoxia (0.1% O_2_) or (d) AZD7762, exhibited no visible tail moment (TM) with TM values ranging from 0.1-0.3. In contrast, cells co-treated with AZD7762 and TH-302 (e-h) exhibited an increased TM, indicating the induction of double-strand breaks (DSBs). Under normoxia (21% O_2_), DSBs induced by co-treatment were TH-302 concentration-dependent (e and f). However, under hypoxia, lower TM values were observed at higher TH-302 concentration (h) than lower concentration (g) due to the superimposition of DNA cross-linking. Quantification of DNA cross-linking was investigated in cells treated with bleomycin for 1 h starting at the end of the drug treatment period. As shown in Figure 3C and D, 20 μM of bleomycin-alone (b) produced profound DNA breaks with a TM of 29 compared with DMSO-treated cells (a). TH-302 alone under normoxia (c), under hypoxia (0.1% O_2_) (d) and AZD7762 alone (e) did not produce visible TM. Consistent with previously published data [4], TH-302 treatment effectively cross-linked DNA under hypoxia (0.1% O_2_; h and i) but not under normoxia (21% O_2_; f and g). The addition of AZD7762 did not change the magnitude of TH-302 cross-linking as the measured TMs (k-n) were not different. AZD7762 alone did not induce detectable DNA cross-linking at the tested concentration (j). The AZD7762 concentration tested (IC_5_) is well-below the IC_50_, and thus may be too low to detect DNA damage.

### AZD7762 increases the TH-302 mediated DNA damage response of γH2AX

**Figure 4:**
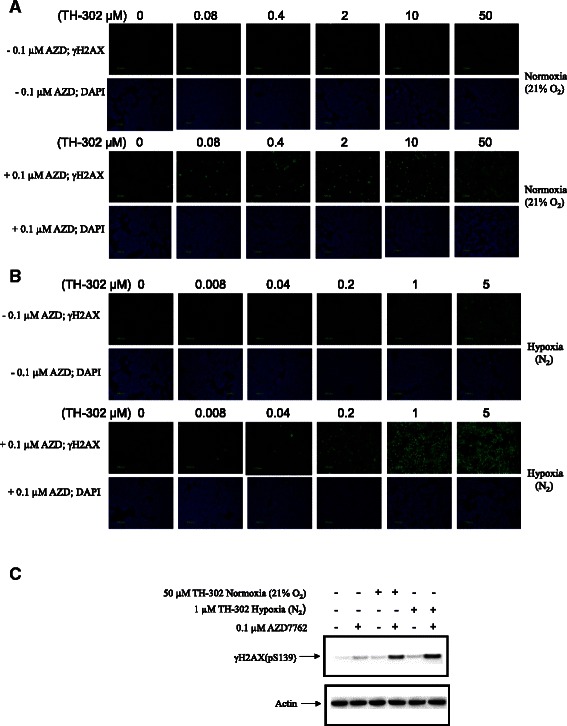
Enhanced γH2AX signal by co-treatment of TH-302 and AZD7762. HT29 cells were treated with TH-302 alone at the indicated concentrations, 0.1 μM AZD7762 alone, or combined treatment of TH-302 and 0.1 μM AZD7762 under either air (21% O_2_) or hypoxia (N_2_) for 2 h and then washed. The signal of γH2AX was detected by immunofluorescence microscopy **(A and B)** after additional 4 h incubation in the presence of 0.1 μM AZD7762 for combination group and AZD7762 group. The blue-fluorescent nucleic acid stain DAPI was used as a nuclear counterstain. Immunoblot **(C)** data were generated after 22 h additional incubation in the presence of 0.1 μM AZD7762 for combination group and AZD7762 group. The data are the representative of two independent experiments.

To investigate the effects of Chk1 inhibition on TH-302-mediated DNA damage response, HT-29 cells were treated with TH-302, AZD7762, or combined TH-302 and AZD7762, and analyzed for γH2AX using fluorescence microscopy (Figure 4A-B). Cells treated with 0.1 μM of AZD7762 under both normoxia (21% O_2_) and hypoxia (N_2_) exhibited minimal induction of γH2AX. Consistent with previously published results [4], TH-302 induced γH2AX in a concentration-dependent and hypoxia-selective manner. Co-incubation with AZD7762 and TH-302 resulted in a marked increase in γH2AX under both normoxia (21% O_2_) and hypoxia (N_2_). Immunoblot analysis of HT29 cells (Figure 4C) showed that variant histone H2AX was only phosphorylated significantly in cells treated with both TH-302 and AZD7762 under either normoxia (21% O_2_), or hypoxia (N_2_) but not cells treated with vehicle, AZD7762, or TH-302 alone under either normoxia (21% O_2_) or hypoxia (N_2_).

### Involvement of DNA repair in Chk1-mediated enhancement of TH-302 cytotoxicity

**Figure 5:**
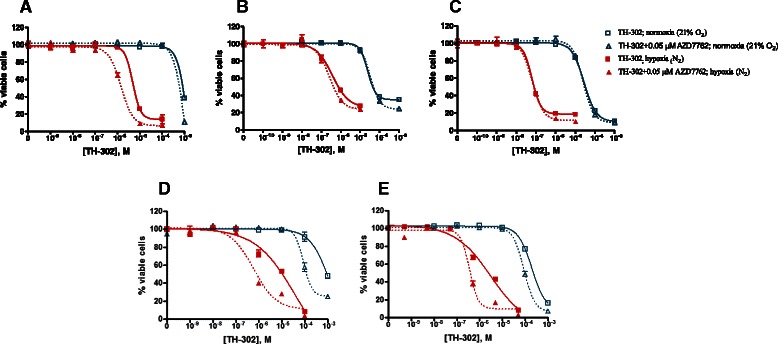
ADZ7762 enhanced TH-302-mediated cytotoxicity only in HDR-proficient but not in HDR-deficient cells regardless of NHEJ status. HDR-proficient CHO AA8 **(A)**, HDR-deficient CHO irs1SF **(B)**, HDR-deficient CHO UV41 **(C)**, NHEJ-proficient CHO K1 **(D)** and NHEJ-deficient CHO xrs5 cells **(E)** were seeded 24 h prior to experiment. After compound addition, cells were incubated for 2 h with TH-302 and 0.1 μM AZD7762 under either normoxia (21% O_2_) or hypoxia (N_2_). After wash, cells were continuously incubated for additional 70 h in complete medium containing 0.1 μM of AZD7762. The viable cells were quantified using AlamarBlue and IC_50_ values were calculated using Prism software. The data are the representative of three independent experiments.

To investigate whether DNA repair processes play a role in the enhancement of TH-302 cytotoxicity by Chk1 inhibitors, we conducted an *in vitro* combination study with TH-302 and AZD7762 using paired HDR-proficient and HDR-deficient cells. Interestingly, Chk1 inhibition enhanced TH-302-mediated cytotoxicity only in HDR proficient CHO AA8 cells and had no significant effect on the cytotoxicity of TH-302 in HDR-deficient and cell-cycle checkpoint-proficient irs1SF and UV41 cells (Figure 5A-C). AZD7762 enhanced TH-302 cytotoxicity equally in either non-homologous end joining (NHEJ)-proficient or NHEJ-deficient cells (Figure 5D-E).

### AZD7762 increases TH-302-mediated apoptosis

**Figure 6:**
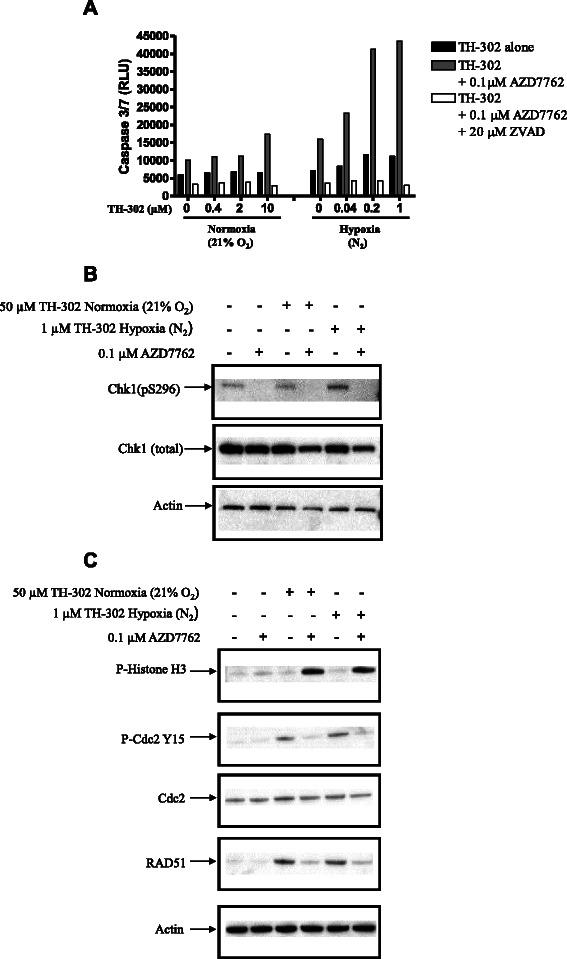
**(A)** AZD7762 enhanced apoptosis in cells co-treated with TH-302. HT29 cells were exposed to TH-302 at the indicated concentrations and 0.1 μM of AZD7762 for 2 h under either normoxia (21% O_2_) or hypoxia (N_2_). After wash, cells were continuously cultured for additional 46 h in the presence of 0.1 μM AZD7762. Caspase 3/7 activity was detected using luminescence-based caspase activity assay kit. The specificity of caspase 3/7 activity was confirmed by including pan-caspase inhibitor ZVAD. **(B)** AZD7762 inhibited Chk1 autophosphorylation. HT29 cells were treated with TH-302, AZD7762, or combination of TH-302 and AZD7762 for 2 h, washed, and AZD7762 added back and incubated for an additional 46 h. Cell lysates were harvested and immunoblotted with antibodies against Chk1 autophosphorylation (S296) and total Chk1. Equal loading was confirmed by actin blot. **(C)** TH-302 AZD7762 co-treatment abrogated cell cycle arrest biomarkers and decreased Rad51 expression. Cells were treated as described in Figure 6B, and then immunoblotted with antibodies against cell-cycle biomarkers phospho-histone H3, phospho-Cdc2 Y15, total Cdc2 and Rad51. Equal loading was confirmed by actin blot. The blot data are the representative of two independent experiments.

To investigate the effect of AZD7762 on TH-302-mediated apoptosis, HT-29 cells were treated with TH-302, AZD7762 or both TH-302 and AZD7762 and analyzed for caspase 3/7 activity. As shown in Figure 6A, TH-302 alone did not induce caspase 3/7 activity under normoxia (21% O_2_) and only slightly increased it under hypoxia (N_2_). Cells treated with AZD7762 alone showed a slight increase in caspase 3/7, especially under hypoxia (N_2_). However, co-treatment of TH-302 and AZD7762 greatly enhanced caspase 3/7 activity. To confirm the specificity of the apoptotic endpoint, the pan-caspase inhibitor ZVAD was employed, which completely abolished caspase 3/7 activity–mediated by drug treatments.

### Effects of TH-302 and Chk1 inhibition on Chk1 signaling pathways

To confirm that AZD7762 inhibits Chk1, we analyzed the status of Chk1 autophosphorylation (S296 Chk1) and total Chk1. As shown in Figure 6B, TH-302 increased Chk1 autophosphorylation but did not affect total Chk1 expression. Consistent with published data [26], nontoxic concentration of 0.1 μM AZD7762 inhibited Chk1 autophosphorylation both at the basal level (lane 2) and in context of enhanced Chk1 autophosphorylation after addition of TH-302 under either normoxia (21% O_2_; lane 4) or hypoxia (N_2_; lane 6) after 48 h AZD7762 treatment. Total Chk1 expression was decreased after combination treatment of TH-302 and AZD7762 although treatment with either TH-302 or AZD7762 alone did not affect total Chk1 expression. Equal loading was confirmed by actin blot. Similar results were also observed after 24 h AZD7762 treatment (data not shown).

As shown in Figure 6C, HT29 cells treated with AZD7762 did not show any alteration in the expression level of phospho-Histone H3 compared with DMSO vehicle-treated cells. Unlike AZD7762, TH-302 under either normoxia (21% O_2_) or hypoxia (N_2_) caused a dramatic decrease of phospho-Histone H3. This effect is indicative of S-phase cell-cycle arrest, which is consistent with the increase S-phase cell population observed after TH-302 treatment (Table 6). The addition of 0.1 μM AZD7762 to TH-302 treatment caused a significant induction of phospho-Histone H3 under normoxia (21% O_2_) or hypoxia (N_2_). Flow cytometry confirmed S-phase checkpoint abrogation with the majority cells having 4 N DNA content (Table 6).

As shown in Figure 6C, treatment of HT29 cells with TH-302 results in an increase of Cdc2-Y15 phosphorylation as a consequence of cell cycle arrest. Addition of the Chk1 inhibitor AZD7762 blocked the TH-302-mediated increase of Cdc2-Y15 phosphorylation, without affecting the expression level of total Cdc2. Cells treated with AZD7762 alone did not show any changes in expression levels of phospho-cdc2 or total cdc2. Similar observations were also observed in HeLa cells (data not shown).

We next tested whether the ability of AZD7762 to sensitize HT29 cells to TH-302 correlate with an inhibition of the DNA damage response through regulation of Rad51 levels, a key protein involved in HDR. As shown in Figure 6C, TH-302 alone increased the expression level of Rad51 by Western blot analysis while AZD7762 alone did not significantly alter the expression level of Rad51. The addition of AZD7762 significantly attenuated the expression of Rad51 in cells co-treated with TH-302. Thus, the expression changes of histone H3, Cdc2-Y15 and Rad51 suggest that the combination increases the DNA damage response, consistent with the increased DNA damage directly measured with the single cell gel electrophoresis comet assay.

### AZD7762 potentiates the anti-tumor efficacy of TH-302 in the HT-29 xenograft model

**Figure 7:**
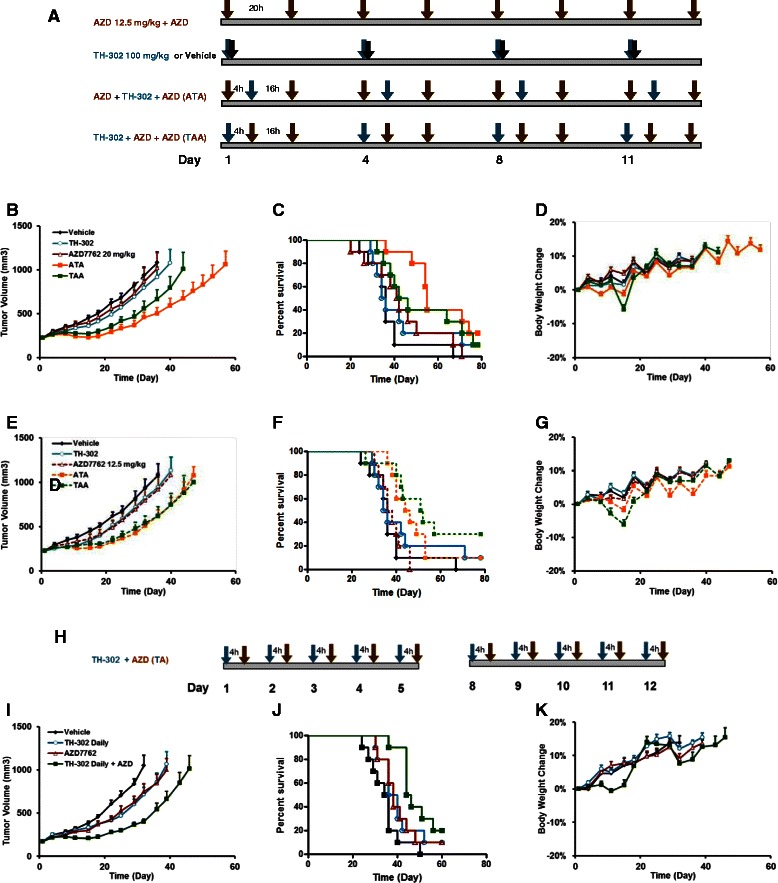
AZD7762 enhanced TH-302 efficacy in HT29 xenograft. Antitumor efficacy of TH-302 alone or in combination with AZD7762 under TH-302 Intermittent Dosing Regimen, presented as study design **(A)**, tumor growth curve **(B, E)**, Kaplan-Meier plot **(C, F)** and body weight change **(D, G)**; TH-302 was given ip at 100 mg/kg, twice a week and AZD7762 was given at 20 **(B, C, and D)** and 12.5 mg/kg **(E, F and G)**, four times a week, respectively. Two dosing sequences, ATA and TAA, were employed in the combination groups. Antitumor efficacy of TH-302 alone or in combination with AZD7762 under TH-302 Daily Dosing Regimen, presented as study design **(H)**, tumor growth curve **(I)**, Kaplan-Meier plot **(J)** and body weight change **(K)**. TH-302 and AZD7762 were given ip daily for 5 days a week at 50 and 12.5 mg/kg, respectively. In the combination group, TH-302 was given 4 hours prior to AZD7762. Data are mean ± SEM of 10 animals per group.

20 mg/kg AZD7762 in combination with 100 mg/kg TH-302 reached MTD. AZD7762 20 mg/kg and 12.5 mg/kg were used. Two dosing sequences of treatment were employed in the combination groups (Figure 7A). In the high dose of AZD7762 20 mg/kg groups, TH-302 or AZD7762 alone exhibited antitumor activity, however, the tumor inhibition observed with the combination of TH-302 and AZD7762 was significantly increased in both dosing sequences tested (p < 0.05, Figure 7B and C, Table 7). There was no statistically significant difference in the tumor growth inhibition observed between the two dosing sequences, but there was a clear trend suggesting that the ATA sequence yielded greater antitumor activity compared with TAA, with TGI increased by 19%. The TGD to 1000 mm^3^ was doubled in the ATA sequence group compared with the TAA group. In addition, using conditional survival as a read out, the ILS was 53% in ATA group vs. 22% in TAA group.

**Table 7:** **Antitumor activity and body weight change of TH-302 in combination with AZD7762 in the HT29 xenograft model**

	TH-302 Intermittent Regimen					TH-302 Intermittent Regimen					TH-302 Daily Regimen				
	TH-302 100 mg/kg, AZD 20 mg/kg,					TH-302 100 mg/kg, AZD 12.5 mg/kg,					TH-302 50 mg/kg, AZD 12.5 mg/kg,				
	TGI	TGD_1000_	MT	ILS	MBL	TGI	TGD_1000_	MBL	MT	ILS	TGI	TGD_1000_	MBL	MT	ILS
	(%)	(days)	(days)	(%)	(%)	(%)	(days)	(%)	(day)	(%)	(%)	(days)	(%)	(days)	(%)
Vehicle			36		0			0	36				0.1	35	
AZD7762	7.7	2	42	15	0	16	3	0	37	28	34	5	0	38	8.6
TH-302	19	4	35	-2.8	0	19	4	0	35	-2.8	38	7	0	38	8.6
ATA	67^*,a,b^	21	55^*,a^	53	1.3	54^*,a,b^	11	1.8	45^*,a^	25	-	-	-	-	-
TAA	48^*,a,b^	10	44^*^	22	5.8	54^*,a,b^	13	6.1	52	43	-	-	-	-	-
TA	-	-	-	-	-	-	-	-	-	-	74^*a,b^	16	0.7	45^*^	29

TGI, tumor growth inhibition.

TGD1000, tumor growth delay to 1000 mm^3^.

MBL, maximal body weight loss due to drug treatment as compared with the first day of treatment.

MT: Median Time to reach the size of 1000 mm^3^.

ILS: Increased Life Span.

^*^p <0.05 vs. vehicle group.

^a^p <0.05 vs. AZD7762 group.

^b^p <0.05 vs. TH-302 group.

Toxicity of the different regimens was assessed by body weight changes. As shown in the Figure 7D, TH-302 or AZD7762 alone did not induce body weight loss. When the two drugs were combined, maximal body weight loss in the ATA sequence group was only 1.3% compared with 5.8% in the TAA group. Taken together with antitumor activity, the results indicate that ATA is a more optimal dosing sequence with a more favorable therapeutic index.

Lower dose of AZD7762 12.5 mg/kg in combination with TH-302 also showed an enhanced antitumor activity (Figure 7E and F). Here the two dosing sequences yielded similar antitumor activity and no dosing sequence superiority was observed between ATA and TAA. In the Kaplan-Meier plot, MT in the ATA group was 45 days compared to 52 days in the TAA group, but there was no significant difference between the two groups. Consistent with the higher dose of AZD7762 experiment, the TAA sequence group experienced more body weight loss than the ATA sequence group (Figure 7G).

In the TH-302 daily dosing regimen study, a similar additive effect was observed in the combination groups, and there was almost no body weight loss observed throughout the study (Figure 7H-K, Table 7).

### Pharmacodynamic biomarkers responsive to AZD7762 and TH-302 in HT29 xenografts

**Figure 8:**
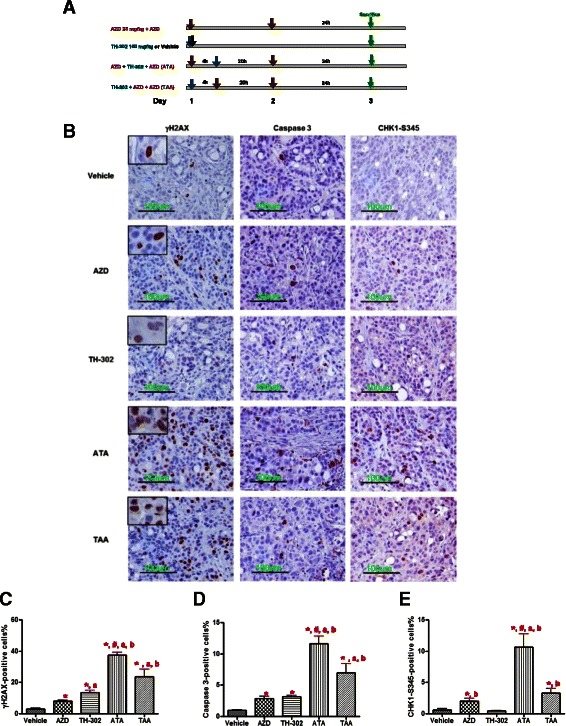
Effects of AZD7762 and TH-302 on pharmacodynamic biomarkers in HT29 xenografts. **(A)** schematic diagram illustrating the study design. **(B)** representative images of γH2AX, Caspase 3 and Chk1-S345 immunostainings, respectively. Insets in γH2AX images, magnification of γH2AX positive cells showing the staining pattern was either pan-nuclear or foci-formation. **(C, D and E)**, morphometric analysis of γH2AX, Caspase 3 and Chk1-S345 positive cells. Scale bar, 100 μm; Each bar represents mean ± SEM of 5 animals per group. * p <0.05 vs. vehicle group; ^a^, p <0.05 vs. AZD7762 group; ^b^, p <0.05 vs. TH-302 group. ^#^, p <0.05 vs. TAA group.

The treatment schedule is presented in Figure 8A. In the vehicle treated HT29 xenograft tumors, few γH2AX positive cells were present (Figure 8B). After AZD7762 or TH-302 treatment, there is a significant increase of γH2AX positive cells (8.2 ± 0.5% and 13.6 ± 0.5% in the AZD7762 and TH-302 groups, respectively, compared with 3.1 ± 0.5% in vehicle, p < 0.05). In the combination groups, the percentage of γH2AX positive cells observed was significantly higher than either monotherapy group (Figure 8C), and γH2AX positive cells after ATA sequence treatment was significantly higher than observed in the TAA sequence group. γH2AX positive cells in vehicle, AZD7762, and combination groups exhibited pan-nuclear staining, while in the TH-302 treated group, foci of γH2AX formation was more commonly observed. Similar results were observed on Caspase 3 immunostaining (Figure 8D), a marker for apoptosis. To confirm Chk1 inhibition by AZD7762 *in vivo*, we analyzed phospho-Chk1-S345 positivity. The number of phospho-Chk1-S345 positive cells increased in response to AZD7762 treatment, but not to TH-302 treatment; and a further increase in the combination treated animals with the greatest increase in the ATA group (Figure 8E).

## Discussion

Both the *in vitro* and *in vivo* preclinical results described here demonstrate the potentiation of TH-302 efficacy by the addition of Chk1 inhibitors in the context of p53 deficiency. Our findings suggest that TH-302 cytotoxicity may be enhanced through at least two Chk1-dependent mechanisms. The first mechanism is abrogation of DNA damage-dependent cell cycle arrest, which is supported by the finding that Chk1 inhibition sensitizes the p53^-/-^ cells but not the p53^+/+^ cells to TH-302. Substantial literature supports that Chk1 inhibitors selectively sensitize tumor cells with p53 deficiency to DNA damaging agents [24-28]. More than 50% of human tumors have mutations in p53 [31]. Cells deficient in p53 cannot undergo p53-dependent apoptosis and are resistant to drugs that induce a p53-dependent apoptosis. Such resistance has been reported in many p53 null or mutated human cancer cell lines as well as in clinical samples [32]. Indeed inhibition of Chk1 in tumor cells, either by siRNA knockdown of Chk1 protein expression [19], or by small molecules inhibiting its kinase activity, demonstrated its role in the potentiation of the cytotoxic activity of DNA damaging agents [18,19] This model is also supported by the findings that Chk1 inhibition preferentially sensitizes p53 deficient human cancer cells, but not p53 functional cells to gemcitabine, radiation and 5-fluorouracil [29,33,34].

The second mechanism of enhanced TH-302 cytotoxicity by Chk1 inhibitor is related to HDR inhibition. Based on our previously published study, HDR plays the key role in the response and repair of TH-302-induced DNA cross-links [4]. Enhanced TH-302 activity was also observed in Rad51 knockout cell lines and in triple-negative breast cancer cell lines exhibiting an HDR-deficient (BRCA-like) phenotype [35,36]. It has been proposed that Chk1 is required for HDR [17], which normally occurs in the S and G_2_ phase [37]. p53-mutated cells lack a G_1_ checkpoint, and thus they may be more dependent on HDR [26]. Thus, it would be anticipated that Chk1 inhibition would predominantly affect HDR in p53-mutated cells [17]. The requirement for HDR inhibition in TH-302 sensitization by Chk1 inhibitors is shown by a lack of TH-302 sensitization by Chk1 inhibition in HDR-deficient cells. However NHEJ-proficient and -deficient cells exhibited a similar Chk1 inhibitor-involved sensitization to TH-302. Furthermore, our findings demonstrate that Chk1 inhibitors can down-regulate TH-302-induced overexpression of Rad51, and subsequently restore cell sensitivity to TH-302. The current findings suggest that Chk1 inhibition may offer considerable benefit to TH-302 in Rad51-overexpressing tumors. Pancreatic ductal adenocarcinoma is a cancer type where overexpression of Rad51 has been described [38]. Chk1 is involved in HDR by directly phosphorylating Rad51 and in the recruiting of Rad51 to sites of DNA damage [17,39,40]. It has also been reported that a Chk1 inhibitor reduces Rad51-mediated HDR [17]. To determine whether Rad51 is involved in the enhancement of TH-302 by AZD7762, we examined the Rad51 expression in cells exposed to TH-302, AZD7762, or combined TH-302 and AZD7762. Rad51 levels increased in cells treated with TH-302. Rad51 protein levels were not affected by AZD7762. However, AZD7762 abolished Rad51 upregulation mediated by TH-302. Although both inhibition of cell cycle arrest and HDR are associated with TH-302 sensitization by Chk1 inhibitors, the relative importance of these effects remains to be determined.

Upon DNA damage, Chks are activated and promote cell cycle arrest at G_2_ phase. G_2_ arrest correlates with an increase of Cdc2 inhibitory phosphorylation at its T14 and Y15 sites. Flow cytometry data confirmed that Chk1 inhibitors abrogate TH-302-induced G_2_ arrest in HeLa cells and S arrest in HT 29 cells. Consistent with flow cytometry data, induction of pY15 Cdc2 was observed following TH-302 treatment *in vitro* and this signal was abolished and phosphorylation of histone H3 was enhanced in the co-treatment group of TH-302 with Chk1 inhibitors.

Co-treatment of TH-302 and AZD7762 caused a dramatic increase in DNA breaks as measured by γH2AX staining and directly assessed with the single cell electrophoresis comet assay. Co-treatment of TH-302 and AZD7762 under normoxia produced a concentration-dependent and a greater tail moment compared to under hypoxia. This observation is consistent with a high level of DNA cross-linking of the broken DNA fragments under hypoxia, leading to slower migration of larger molecular weight fragments.

Chk1 plays a key role in protecting cells from apoptosis in response to many types of DNA damage [21]. Down-regulation of Chk1 has been shown to selectively induce apoptosis in cancer cells [41]. Our data showed that co-treatment of TH-302 and AZD7762 induced apoptosis although neither TH-302 nor AZD7762 alone induced a high level of apoptosis.

Preclinical studies have shown that AZD7762 potentiates DNA and replication-targeted therapies, including cisplatin, gemcitabine, irinotecan, and paclitaxel [24,29,42-44]. Similarly, the antitumor efficacy and levels of the corresponding biomarkers, γH2AX and caspase-3, from the combination of TH-302 and AZD7762 was significantly increased in the p53 mutant HT29 human tumor xenograft model. γH2AX was induced by AZD7762 or TH-302 alone and to a greater level when AZD7762 and TH-302 were combined. The induction of γH2AX by AZD7762 alone was consistent with the findings of Mitchell *et al.* [24], and which may be the result of replication stress [18,45]. Of note, the higher dose AZD7762 (20 mg/kg) group showed a sequence-dependent superior efficacy when combined with TH-302, which was consistent with the biomarker findings showing enhanced downstream PD effects with the same sequence. AZD7762 given first, followed by TH-302, and then followed by another dose of AZD7762 might be an optimal dosing sequence for future preclinical and clinical studies. Taking together the *in vivo* efficacy profile and pharmacodynamic results, the HT29 xenograft data reported here support the hypothesis for a selective enhancement of TH-302 antitumor activity by the co-administration of a Chk1 inhibitor. As TH-302 predominantly and selectively targets hypoxic cells [4,6] even though all the tumor cells are chemosensitized by AZD7762, a triplet combination of AZD7762, TH-302, and a chemotherapeutic targeting the normoxic compartment may lead to an even superior efficacy profile [5]. A similar therapeutic strategy can also be applied with hypoxia-activated Chk1 inhibitors [15,16] in combination with conventional cancer drugs targeting normoxic cells.

The results presented here support the following model for enhanced TH-302 activity by Chk1 inhibition. Hypoxia-activated TH-302 fragments in a one-electron reductase-dependent and hypoxia-selective manner, and releases the bis-alkylating effector Br-IPM, causing DNA cross-linking. Upon the recognition of the DNA damage by the DNA damage response (DDR), the variant histone H2AX is phosphorylated; yielding γH2AX, and Chk1 is activated. Chk1 phosphorylates Cdc2 at Y15, blocking phosphorylation of histone H3 and arresting the cell cycle at G_2_/M and S phase as well as allowing cells to undergo HDR-mediated DNA repair. When Chk1 is inhibited by a small molecule inhibitor, for example AZD7762, Chk1 inhibition triggers phosphorylation of histone H3, reduces Cdc2 phosphorylation, and leads to abrogation of cell cycle arrest. This allows arrested cells to progress directly into mitosis in cells co-treated with TH-302. Furthermore, Chk1 inhibition downregulated TH-302-induced upregulation of DNA repair protein Rad51, a key component of the specific DNA repair pathway. TH-302 treatment in the context of Chk1 inhibition activates caspases and induces apoptosis.

## Conclusions

In conclusion, we have demonstrated that TH-302 *in vitro* and *in vivo* activity were greatly enhanced by Chk1 inhibition. The data presented here could have relevance to the clinical use of TH-302 by combining with Chk1 inhibitors for the treatment of p53-deficient cancers.

## Abbreviations

Br-IPM: Bromo-isophosphoramide mustard
Chk: Check-point kinase
DSBs: Double-strand breaks
HDR: Homology-directed repair
NHEJ: Non-homologous end joining
MT: Median time
TGD: Tumor growth delay
TGI: Tumor growth inhibition
TM: Tail moment
ZVAD: Benzyloxycarbonyl-Val-Ala-D
DDR: DNA damage response
PD: Pharmacodynamic
